# Development of Dot-ELISA and Colloidal Gold Immunochromatographic Strip for Rapid and Super-Sensitive Detection of Plum Pox Virus in Apricot Trees

**DOI:** 10.3390/v15010169

**Published:** 2023-01-05

**Authors:** Mengmeng Guo, Duo Qi, Jinxi Dong, Saiyu Dong, Xiuling Yang, Yajuan Qian, Xueping Zhou, Jianxiang Wu

**Affiliations:** 1 State Key Laboratory of Rice Biology, Key Laboratory of Biology of Crop Pathogens and Insects of Zhejiang Province, Institute of Biotechnology, Zhejiang University, Hangzhou 310058, China; 2 Hainan Institute, Zhejiang University, Sanya 572025, China; 3 State Key Laboratory for Biology of Plant Diseases and Insect Pests, Institute of Plant Protection, Chinese Academy of Agricultural Sciences, Beijing 100193, China

**Keywords:** apricot tree, plum pox virus, monoclonal antibody, dot-ELISA, colloidal gold immunochromatographic strip, RT-PCR

## Abstract

Plum pox virus (PPV) is a causal agent of the stone fruit tree sharka disease that often causes enormous economic losses. Due to its worldwide distribution and economic importance, rapid and reliable diagnostic technologies are becoming increasingly important for successful management of sharka disease. In this study, we have produced two super-sensitive and specific anti-PPV monoclonal antibodies (i.e., MAbs 13H4 and 4A11). Using these two MAbs, we have now developed a dot enzyme-linked immunosorbent assay (dot-ELISA) and a colloidal gold immunochromatographic strip (CGICS) assay. These two technologies can be used to quickly and reliably detect PPV. The results of these sensitivity assays confirmed that the dot-ELISA and CGICS assays could detect PPV infection in apricot tree leaf crude extracts diluted up to 1:5120 and 1:6400 (*w*/*v*), respectively. Further analyses using field-collected apricot tree leaf samples showed that the detection endpoint of the dot-ELISA was ~26 times above that obtained through RT-PCR, and the CGICS was as sensitive as RT-PCR. This present study is to broaden the knowledge about detection limits of dot-ELISA and CGICS for PPV monitoring. We consider that these newly developed dot-ELISA and CGICS are particularly useful for large scale PPV surveys in fields.

## 1. Introduction

Plum pox virus (PPV) belongs to the genus *Potyvirus*, family *Potyviridae*. Because PPV can cause severe sharka disease, it is considered as the most devastating virus of stone fruit trees worldwide [1]. PPV can infect numerous *Prunus* species, including plum, apricot, peach, nectarine, almond, sweet cherry, and many other important ornamental species [2], and causes huge economic losses [3]. Sharka disease was first documented in plum trees in 1915 in Bulgaria and reported in 1932. This disease was then found in apricot trees in 1933 [4]. Despite significant efforts made by farmers and research communities, this disease has spread into at least 55 countries in Europe, Africa, Asia, and America [5] (https://www.cabi.org/isc/datasheet/42203, accessed on 20 October 2022).

PPV-infected trees often show disease symptoms in leaves and fruit. In spring, the PPV-induced symptoms are often conspicuous, including yellow rings, diffused chlorotic spots, and vein-clearing in plum and apricot tree leaves [3]. PPV-infected fruits also show chlorotic spots, brownish or reddish necrotic flesh, or fall prematurely. PPV-infected apricot kernels often show discolored rings or spots [6]. PPV is transmitted in the field by many aphid species, such as *Aphis spiraecola*, *Aphis gossypii*, *Myzus persicae*, *Phorodon humuli*, and *Hyalopterus pruni*, in a non-circulative manner [7,8,9]. PPV can also be transmitted through grafting in nurseries [10,11]. Therefore, transport of infected plant materials and tree seedings from one location to another is often considered as the main route of PPV long-distance spread, including the spread between two different countries.

PPV has a positive-sense, single-strand RNA genome of approximately 9.8 kb in length. The 5′ end of the PPV genome is covalently bound by a viral-linked protein (VPg) and its 3′ end has a poly (A) tail [12]. PPV virions are filamentous and are approximately 750 nm in length and 15 nm in diameter [2]. To date, ten PPV strains have been reported according to viral genome sequences, and biological and epidemiological characters. These strains are PPV-D, PPV-M, PPV-EA, PPV-C, PPV-Rec, PPV-W, PPV-T, PPV-CR, PPV-AM, and PPV-CV [13]. PPV-D is currently the most common strain in Europe, North America, South America, and Africa [14]. In the past two decades, many PPV-D isolates have been reported in the East and South Asia countries [14]. The PPV-D isolates found in Japan have been considered to come from overseas and are now widely spread in commercial Japanese apricot trees (*Prunus mume*) [15,16,17]. The PPV isolates found in South Korea are evolutionarily clustered closely with the isolates of PPV-D from Japan [18]. In 2005, PPV was first reported in apricot trees in the Hunan Province, China [19], and then found in the Japanese apricot trees grown in the Jiangsu Province, China in 2007 [20]. In 2007, PPV became a pathogen in the list of Imported Plant Quarantine Pests of China. Field surveys conducted in stone fruit tree farms from 2008 to 2018 have found that PPV is now widely distributed in the common apricot (*P. armeniaca*) and Japanese apricot trees in China [6]. Phylogenetic analysis has shown that all the PPV isolates found in China belong to the PPV-D strain [6]. 

Because PPV is effectively transmitted through infected plant materials and aphids, the best way to control this disease is to eliminate infected trees and to quarantine the plant materials before being transported from one local and commercial nursery to other places using highly specific and sensitive detection methods. To date, several serological and RT-PCR-based methods have been reported for PPV detections [21,22,23,24,25,26,27]. These methods include the double antibody sandwich enzyme-linked immunosorbent assay (DAS-ELISA) [22], triple antibody sandwich ELISA (TAS-ELISA) [23,24], reverse transcription-polymerase chain reaction (RT-PCR) [25], real time RT-PCR [25], reverse transcription loop-mediated isothermal amplification (RT-LAMP), immunocapture RT-PCR (IC-RT-PCR) [26,27], and reverse transcription recombinase polymerase amplification (RT-RPA) [28]. Because these methods are all time-consuming and require complicated operations and expensive laboratory instruments, they are not suitable for rapid and large scale field surveys. 

A colloidal gold immunochromatography strip (CGICS) test is an antibody-based sensitive serological method and can be used in large scale field surveys without the need of expensive equipment and reagents. Thus, this test has become the most frequently used method for detections of viruses in large numbers of field-collected samples [29,30]. Both polyclonal antibody (PAb) and monoclonal antibody (MAb) can be used in the CGICSs for PPV detection [14]. To optimize the sensitivity and reliability of the CGICS, we prepared two super-sensitive and specific MAbs using purified PPV virions as the immunogen. We then used them to develop a dot-ELISA and a CGICS for PPV detection. Because these two assays are super-sensitive and specific, we consider that these two assays are particularly useful for large scale PPV surveys in stone fruit farms.

## 2. Materials and Methods

### 2.1. Virus Sources, Virion Purification

Japanese apricot tree leaves showing PPV-like symptoms were collected from the Nanjing Botanical Garden, Nanjing, China, in 2021. PPV infection in this sample was confirmed through RT-PCR followed by DNA sequencing. PPV virions were then extracted from the collected leaves through differential centrifugation as described previously [31]. The purified virions were negatively stained with 0.1% phosphotungstic acid and examined under an electron microscope to view virion morphology.

### 2.2. Generation and Characterization of Murine Monoclonal Antibodies

Purified PPV virions were used to immunize eight-week-old BALB/c female mice through intraperitoneal injections as previously described [31,32]. Briefly, BALB/c mice was immunized twice with purified PPV virions (80 µg/mouse) emulsified in the complete and incomplete Freund’s adjuvant (Sigma-Aldrich, St. Louis, MO, USA) at a 3-week interval. For the third boost immunization, the purified PPV virions were diluted in a saline solution and injected (100 µg/mouse) intraperitoneally into the mice. Three days later, splenocytes were isolated from the immunized mice and fused with murine myeloma Sp2/0 cells using the polyethylene glycol (MW 3350, Sigma-Aldrich) method as described [31,33]. The fused cells were cultured and then selected on the HAT selection medium (RPMI-1640 medium, Sigma-Aldrich) supplemented with 100 µmol/L hypoxanthine, 0.4 µmol/L aminopterin, 16 µmol /L thymidine, and 10% fetal calf serum (Hangzhou Jiangbin Biotechnology Co., LTD, Hangzhou, China). Supernatant from individual hybridoma cultures was screened for PPV antibody production using an indirect-ELISA [31,33]. Hybridomas secreting anti-PPV MAbs were cloned using the limiting dilution method. The resulting hybridomas were injected intraperitoneally into BALB/c mice to induce ascitic fluids containing MAbs. The MAbs in the ascitic fluids were purified using a saturated ammonium sulfate precipitation method. The specificity and sensitivity of each MAb were determined through Western blot and dot-ELISA assays [32,34]. The titers of the MAbs were determined through an indirect-ELISA using purified PPV virions as the coating antigen. Isotypes of the MAbs were determined using a mouse MAb isotyping kit as instructed (Sigma-Aldrich).

### 2.3. Dot-ELISA

Detection of PPV in leaf samples using dot-ELISA was performed as described previously [31]. Briefly, each apricot tree leaf sample (about 50 mg) was ground in 1 mL of 0.01 mol/L phosphate buffered saline (PBS), pH 7.4, followed by 3 min centrifugation at 5000 rpm at 4 °C. The resulting supernatant was used for PPV detection. A known PPV-infected and uninfected apricot tree leaf samples were used as the positive and negative controls. Each supernatant (2 µL) was loaded onto a nitrocellulose membrane (GE Healthcare, Bucks, UK) and the dotted nitrocellulose membrane was air-dried for 10 min at room temperature (RT). The nitrocellulose membrane was blocked for 30 min in a PBS solution containing 5% skimmed milk powder followed by 1 h incubation in a diluted anti-PPV MAb solution (first antibody). After three rinses in the PBS solution containing 0.05% Tween-20 (PBST), the membranes were probed again for 1 h in a diluted alkaline phosphatase (AP)-conjugated goat anti-mouse IgG (second antibody) followed by three rinses in the PBST. Color reaction on the membrane was visualized after the addition of a nitro-blue tetrazolium chloride/5-bromo-4-chloro-3-indolyl phosphate substrate solution (Sigma-Aldrich) for 10-20 min at RT.

### 2.4. Preparation of Colloidal Gold Nanoparticle-Conjugated Antibody

Colloidal gold nanoparticles (CGNPs), 40 nm in diameter, were prepared using a citrate reduction method as described previously [30,35,36]. The CGNP-conjugated MAbs were made through mixing 1.0 mg of a purified MAb slowly with 60 mL of CGNP solution. The mixture was stirred gently for 30 min at RT followed by the slow addition of 1 mL of 10% bovine serum albumin (BSA) in a 0.01 mol/L borate solution. The mixture was then gently stirred for 30 min at RT followed by 20 min centrifugation at 20,000 *g* at 4 °C. The CGNP-conjugated MAb pellets were rinsed twice with a 0.02 mol/L PBS, pH 7.4, containing 2% BSA, 5% sucrose, and 0.5% polyethylene glycol 6000, and then dissolved in 10 mL of 0.02 mol/L PBS, pH 7.4, containing 3% sucrose, 2% BSA, and 0.02% NaN3. The final CGNP-conjugated MAb was stored at 4 °C until use.

### 2.5. Development of a Colloidal Gold Immunochromatographic Strip (CGICS)

**Figure 1:**
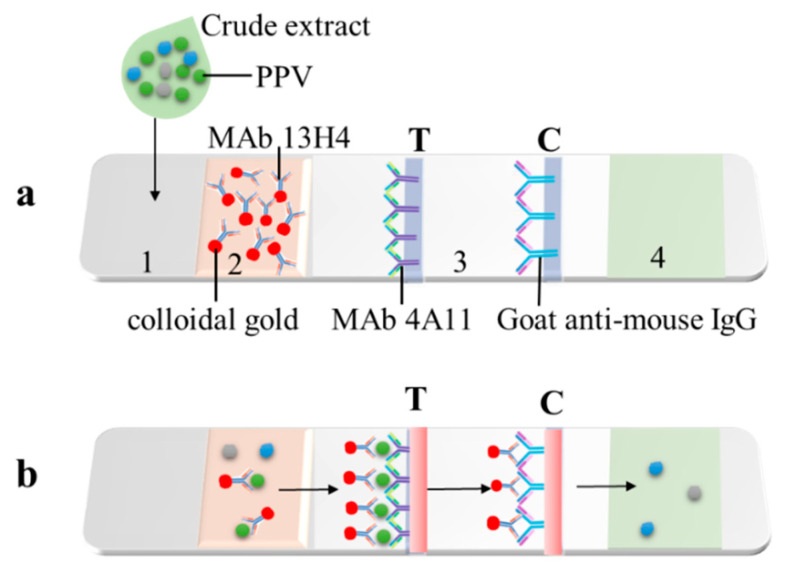
A schematic diagram showing the colloidal gold immunochromatographic strip. (**a**) Designs of CGICS strips. (1) a sample loading pad; (2) a CGNP-MAb 13H4 conjugate pad; (3) a nitrocellulose membrane with MAb 4A11 (**left**) and goat anti-mouse IgG antibody (**right**) at the testing (T) and control (C) line, respectively; and (4) an absorption cellulose fiber membrane pad. (**b**) Diagram showing working principle of the strip. Crude extracts from tree leaf samples are individually dropped to the sample pad. If a sample is infected with PPV, the CGNP-conjugated MAb 13H4 will bind to PPV virions (green dots) at the conjugate pad. The complex migrates to the nitrocellulose membrane and is captured by anti-PPV MAb 4A11 at the T line to form a first red band, whereas the free CGNP-conjugated MAb 13H4 passes through the T line and then is captured by goat anti-mouse IgG second antibody at the C line to form a second red band. The remaining sample will move and accumulate in the absorption pad. The grayish and blue dots represent different plant proteins in the samples.

CGICS was made using the method described previously (30). Briefly, PPV MAb 4A11 and goat anti-mouse IgG were added separately onto a nitrocellulose membrane at the test (T) and control (C) line (Figure 1a, Position 3), respectively, using a Quanti 3000 BioJets attached to a BioDot XYZ-3000 dispensing platform (Bio-Dot, Irvine, CA, USA). The membrane was air-dried at 37 °C for 1 h and then incubated in a PBS solution with 2% BSA for 1 h. The membrane was rinsed three times in the PBST and air-dried for 1 h at 37 °C. The sample and conjugate pad (Figure 1a, Position 1 and 2) was cut from a glass fiber membrane, soaked in a PBS solution, pH 7.4, with 2% BSA, 3% sucrose, 0.05% Tween-20, and 0.05% NaN_3_, and dried at 37 °C for 24 h. The CGNP-conjugated PPV MAb 13H4 was dispensed to the above-treated conjugate pad followed by 2 h incubation at 37 °C. The absorbent pad (Figure 1a, Position 4) was cut from a cellulose fiber membrane, soaked in the PBST, and then dried at 37 °C for 2 h. The sample pad, the CGNP-MAb conjugate pad, the coated membrane, and absorbent pad were laminated orderly and tandemly with 2 mm overlap at the conjunction site and pasted onto the backing plate (Figure 1). The assembled plate was cut longitudinally with a guillotine cutter to produce strips (60 mm long × 3 mm wide). The strips were packaged inside aluminum foil bags with silica gels and stored at RT under dry and lucifugal conditions until use.

### 2.6. Test Procedure of the CGICS

Apricot tree leaf samples (50 mg each) were ground individually in 1 mL of 0.01 mol/L PBS solution, pH 7.4, and the crude extracts (50–150 µL per sample) were dropped individually into the sample pad. After 5–10 min, the samples showing two red bands at the T and C lines were considered as PPV positive, while the samples showing only one red band at the C line were considered as PPV negative. If the C line did not show a red color band, the test was considered as invalid.

### 2.7. Detection of PPV Infection through RT-PCR

To validate PPV infection, total RNA was extracted from the assayed samples using Trizol reagent (Invitrogen, Carlsbad, CA, USA). Concentrations and qualities of the total RNA samples were determined using a Nanodrop Spectrophotometer. The total RNA was reverse-transcribed with the gene-specific reverse primer and the HiScript Reverse Transcriptase kit (Vazyme, Nanjing, Jiangsu, China) according to the manufacturer’s instructions. The subsequent PCR was conducted using a set of conserved PPV primers [PPV-F (5′-CAGACTACAGCCTCGCCAGA-3′) and PPV-R (5′-ACCGAGACCACTACACTCCC-3′)] or a set of PPV-D strain specific primers [PPV-D-F (5′-AGAACCGCCAAGTCAGTA-3′) and PPV-D-R (5′-CATCCAAGTGCCGAACAT-3′)]. Each PCR reaction contained 12.5 μL of 2× Green Taq mix (Vazyme), 1 μL of each primer (10 μmol/L), 1 μL of cDNA, and 9.5 μL of sterile deionized water. The reaction cycle was set at 94 °C for 2 min; 30 cycles of 94 °C for 30 s, 56 °C for 30 s, and 72 °C for 30 s. The final extension was 10 min at 72 °C.

## 3. Results

### 3.1. Preparations of PPV Virions and PPV MAbs

**Figure 2:**
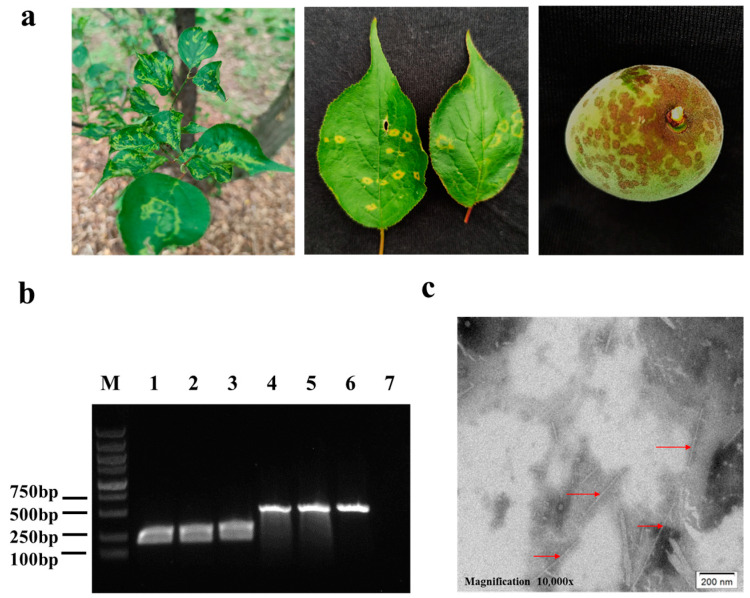
Disease symptoms, RT-PCR detection of PPV infection, and virion morphology. (**a**) Disease symptoms on Japanese apricot tree leaves and fruit. Yellowish ringspots, mosaic, leaf distortion, and a deformed fruit with ringspots and necrotic areas are shown. (**b**) RT-PCR detection of PPV infection in Japanese apricot tree leaf samples. Lane M is a 1 kb DNA ladder. Lane 1–3 represent three Japanese apricot tree leaf samples with PPV-like symptoms. The 243 bp product band were obtained through PCR using the conserved PPV primer pair PPV-F and PPV-R. Lane 4–6 represent three Japanese apricot tree leaf samples with PPV-like symptoms. The 558 bp product band was obtained through PCR using the PPV-D strain specific primer pair PPV-D-F and PPV-D-R. Lane 7 represents a Japanese apricot tree leaf sample without virus-like symptoms (the negative control). (**c**) An electron micrograph showing PPV-like virions. Bar, 200 nm.

Japanese apricot tree leaves showing typical PPV-induced chlorotic ringspots (Figure 2a) were collected from the Nanjing City, Jiangsu Province. RT-PCR results showed that these tree leaf samples were infected with PPV-D strain (Figure 2b). These leaf samples were then used to prepare PPV virions. Under a transmission electron microscope, the purified virion sample was found to have filamentous particles of about 750 nm in length and 15 nm in diameter, similar to potyvirus virions (Figure 2c).

Two murine hybridoma lines (i.e., 13H4 and 4A11) were then prepared using the purified PPV virions as the immunogen. Isotype and subclass analysis results showed that these two MAbs belonged to IgG1, κ light chain. Indirect ELISA result showed that the titers of the two MAbs were up to 10^−7^. The concentrations of IgG in the 13H4 and 4A11 ascites were 7.15 and 6.58 mg/mL, respectively.

### 3.2. Characterization of MAbs 13H4 and 4A11, and Detection of PPV Using Dot-ELISA

**Figure 3:**
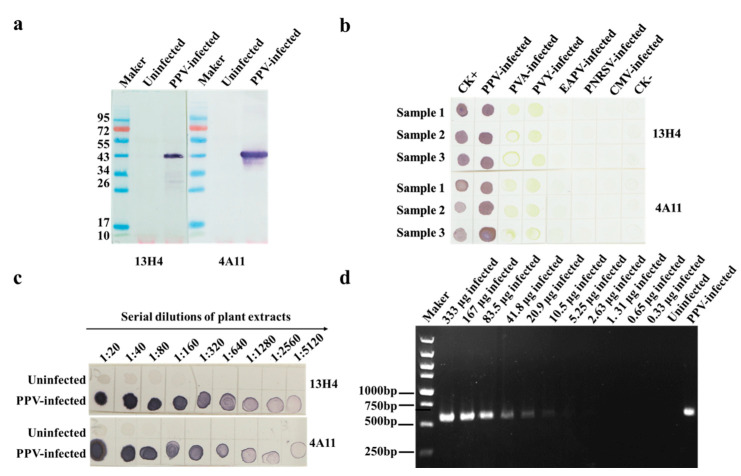
Specificity and sensitivity analyses of the two MAbs through Western blot assay and dot-ELISA. (**a**) Specificity analyses of the two MAbs (13H4 and 4A11) through Western blot assay using a PPV-infected and an uninfected Japanese apricot tree leaf extract. (**b**) Specificity analyses of the two MAbs (13H4 and 4A11) through dot-ELISA using crude extracts from PPV-, PVA-, PVY-, EAPV-, PNRSV-, and CMV-infected leaf samples, respectively. Known PPV-infected Japanese apricot (CK+) and uninfected Japanese apricot tree leaf tissues (CK-) were used as the positive and negative controls. (**c**) Sensitivity analyses of the two MAbs (13H4 and 4A11) through dot-ELISA. The PPV-infected and uninfected extracts were two-fold serially diluted, respectively, in the extraction buffer. Each dilution (2 µL) was loaded onto two nitrocellulose membranes and detected by the two MAbs (13H4 and 4A11), respectively. Purple dots indicate positive reactions, while green or no color dots indicate negative reactions. (**d**) Specificity and sensitivity evaluations of the one-step RT-PCR. Total RNA (30 µL) from 100 mg of PPV-infected Japanese apricot tree leaves was two-fold serially diluted from 1:10 to 1:20,480 (*v*/*v*) in DNase/RNase free ddH_2_O, and the diluted RNA samples (1 μL each) were used for the assay.

Western blot assay was performed to determine the specificities of MAbs 13H4 and 4A11. The results showed that both MAbs reacted specifically with PPV in an infected Japanese apricot tree leaf sample and produced a single protein band (~40 kDa) on the blots. No such protein band was found in the lanes using an uninfected Japanese apricot tree leaf sample (Figure 3a). The molecular weight of the detected protein matched the known molecular weight of PPV capsid protein (CP); thus, we suggest that both MAbs 13H4 and 4A11 can recognize PPV CP.

The results of tree phalanx assays revealed that PPV in the infected apricot tree leaf extracts could be accurately detected through dot-ELISA using 1:5000 (*v*/*v*) diluted 13H4 or 4A11 MAb (primary antibody) and 1:8000 (*v*/*v*) diluted AP-conjugated goat anti-mouse IgG (second antibody). To further confirm the specificities of these two MAbs, we performed dot-ELISA using five plant virus-[potato virus A (PVA), potato virus Y (PVY), East Asian passiflora virus (EAPV), prunus necrotic ring-spot virus (PNRSV), and cucumber mosaic virus (CMV)] infected plant tissues as samples. In this assay, a PPV-infected and an uninfected apricot tree leaf sample were used as the positive and negatives controls, respectively (Figure 3b). The result showed that only PPV was detected in this assay using MAb 13H4 or 4A11, suggesting that these two MAbs are indeed PPV specific.

Sensitivities of the two MAbs were also tested in this study through the dot-ELISA described above using two-fold serially diluted PPV-infected and uninfected Japanese apricot tree leaf samples. The results showed that the PPV detection endpoints using these two MAbs were up to 1:5120 (*w*/*v*, g/mL) dilution for PPV-infected tree leaf crude extracts, equivalent to 0.39 μg of PPV-infected leaf tissues (Figure 3c). To compare the detection sensitivity between dot-ELISAs developed in this study and conventional RT-PCR, total RNA (30 μL) extracted from 100 mg of PPV-infected tree leaves was two-fold serially diluted from 1:10 to 1:20,480 (*v*/*v*) in DNase/RNase free ddH_2_O, and 1 μL of each RNA dilution was subsequently used to determine the sensitivity of one-step RT-PCR. The results showed that PPV could be detected in the 1:320 (*v*/*v*) diluted total RNA isolated from PPV-infected leaves or in 10.5 μg of infected tree leaf tissues (Figure 3d). Surprisingly, the detection limit of the developed dot-ELISA was ~26 times higher than that of RT-PCR, which broadened our knowledge about the detection limit of dot-ELISA for PPV monitoring.

### 3.3. Development of the CGICS for PPV Detection

Our preliminary test showed that the intensity of the detection signal was positively correlated with the concentration of MAb 4A11 used. Because the uninfected control sample produced a weak red band at the T line position when the concentration of MAb 4A11 was above 1.5 mg/mL, we considered that 1.5 mg/mL MAb 4A11 was the optimal concentration for this test. To determine the optimal concentration of the CGNP-conjugated MAb solution, we diluted it in different volumes of 0.02 mol/L PBS (pH 7.4) containing 3% sucrose, 2% BSA, and 0.02% NaN_3_. The test result showed that the 1:30 (*v*/*v*) diluted CGNP-conjugated MAb solution produced a weak false-positive reaction at the T line, whereas the 1:60 diluted CGNP-conjugated MAb solution reduced the detection limit of the strip. Therefore, we considered the 1:50 diluted CGNP-conjugated MAb solution was best for the test.

### 3.4. Specificity and Sensitivity of the CGICS for PPV Detection

**Figure 4:**
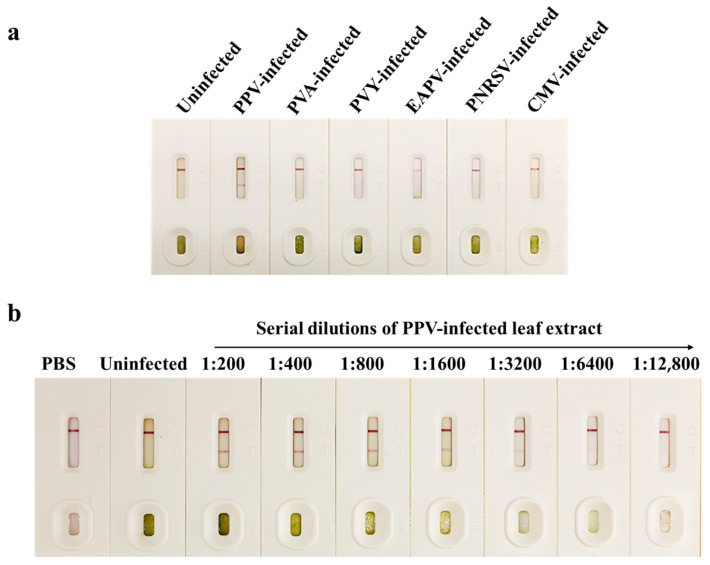
Specificity and sensitivity analyses of the CGICS. (**a**) Specificity of the CGICS was determined using crude extracts from a PPV-infected, other five virus-infected, and an uninfected tissue sample, respectively. (**b**) Sensitivity of the CGICS was determined using serially diluted PPV-infected leaf crude extract. The crude extract from an uninfected Japanese apricot tree leaf sample was used as the negative control. In this test, the 1:200 to 1:6400 diluted extracts produced a red band at the T line.

To determine the specificity of the CGICS, crude extracts were made from a PPV-infected and an uninfected Japanese apricot tree leaf sample, respectively. Crude extracts of an additional five virus-infected leaf samples were also included in this study. The strip test result showed that the PVA-, PVY-, EAPV-, PNRSV-, and CMV-infected samples, and the uninfected control sample all produced a red band at the C line. As expected, the PPV-infected sample produced two red bands at both T and C lines (Figure 4a), indicating that this CGICS can be used to detect PPV infection in plant tissues specifically. To further determine the detection limit of this strip, we serially diluted the crude extracts from a PPV-infected and an uninfected Japanese apricot tree leaf sample in a 0.01 mol/L PBS (1:200 to 1:12,800, *w*/*v*). The diluted samples (60 µL each) were used in this test. The result showed that this CGICS could consistently detect PPV infection in the infected crude extracts diluted up to 1:6400 (*w*/*v*). Therefore, the detection limit of this strip was considered as 1:6400 (*w*/*v*), equivalent to about 9.6 µg PPV-infected leaf tissue (Figure 4b). It is noteworthy that the detection limit of CGICS is the same as that (10.5 µg infected tissues) of one-step RT-PCR performed in this study (Figure 3d).

### 3.5. Detection of PPV Infection in Field-Collected Japanese and Common Apricot Tree Leaves

**Figure 5:**
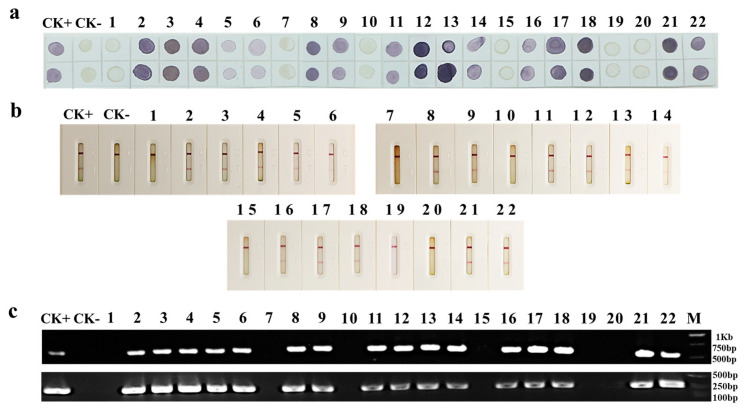
Detection of PPV infection in field-collected tree leaf samples through the newly developed dot-ELISA, CGICS, and RT-PCR, respectively. (**a**) A do-ELISA test using two dots per sample on the same membrane and MAb 13H4 as the detection antibody. Field-collected Japanese apricot tree leaf samples (samples 1 to 19) and common apricot tree leaf samples (samples 20–22) were tested for PPV infection. CK+ and CK− were a known PPV-infected and a known uninfected Japanese apricot leaf sample, respectively. (**b**) The CGICS test of field-collected samples, the same as in Figure 5a. (**c**) RT-PCR analyses using a conserved PPV primer pair PPV-F and PPV-R (**lower** panel) or the PPV-D strain specific primer pair PPV-D-F and PPV-D-R (**upper** panel). The RT-PCR products were about 250 bp and 600 bp, respectively.

To determine the usefulness of the newly developed dot-ELISA and CGICS for PPV detection, we collected 19 Japanese and 3 common apricot tree leaf samples showing PPV-like disease symptoms from seven cities in China (i.e., Beijing, Shanghai, Wuhan, Nanjing, Wuxi, Hangzhou, and Yuncheng) in 2021 and 2022. The do-ELISA result showed that 16 of the 22 samples were infected with PPV (Figure 5a). This result agreed with the result obtained through the CGICS test (Figure 5b). To validate this finding, we analyzed these 22 field samples through RT-PCR using primer set PPV-F and PPV-R or PPV-D-F and PPV-D-R. The RT-PCR results confirmed that the 16 PPV positive samples identified through CGICS and dot-ELISA were indeed infected with PPV (Figure 5c). The other six samples plus the uninfected control sample did not give positive products (Figure 5c). The result of RT-PCR using the PPV-D strain specific primer pair also demonstrated that the 16 positive samples were infected with PPV-D strain (Figure 5c, upper panel).

## 4. Discussion

PPV is the most devastating pathogen of stone fruit trees [1] and is a quarantine pathogen in China [6]. A recent report has shown that the PPV isolates found in the Japanese and common apricot trees in six Chinese cities (Beijing, Nanjing, Shanghai, Wuxi, and Wuhan) belonged to the PPV-D strain [6]. It is well known that PPV invades a new geographic region through introductions of infected stone fruit tree materials. Once the initial infection is established, PPV can be transmitted to surrounding trees by aphids in a non-persistent manner. This transmission chain can be effectively blocked through early detection and quick elimination of infected trees. Thus, establishment of an easy-to-use and reliable quarantine technology for PPV detection in stone fruit trees is crucial in management of sharka disease in China. In addition, this quarantine technique can also help large-scale productions of PPV-free seedlings in stone fruit tree nurseries.

PPV detection techniques have had significant improvements in the past few decades, starting from biological tests using indicator plants to serology assays, and then to molecular technologies [14,21,22,23,24,25,26,27,28]. Because serological methods are easy to use, cost effective, and most results obtained through serological tests agree with the results obtained through PCR or RT-PCR, serological methods are often considered as the most economical and reliable methods for plant virus detections, including PPV [14,37]. During serological tests, polyclonal antibodies (PAbs) can recognize multiple epitopes on viral proteins or virions and thus, the specificity of PAbs always create controversy [38]. On the other hand, MAbs recognize only a single epitope on viral proteins or virions. Thus, high quality MAbs are now widely used in virus detections [38,39]. PCR-based virus detection methods and ELISA are also widely used in plant virus detections but require expensive equipment. Therefore, these two methods are not accessible to farms and local laboratories. In addition, these two methods require more time to complete. CGICS is a rapid, easy-to-use, and cost-effective serological method. This method is particularly useful for large-scale field surveys. In 2010, Byzova and others reported an immunochromatographic strip assay for PPV detection [40]. Maejima and others have also reported an immunochromatographic strip using the PAb against the recombinant expressed CP of a PPV-D isolate, and the detection limit of the strip was up to 1:512 dilution for PPV-infected leaf crude extracts [14]. The commercial immunochromatographic strip made by Agdia Inc. (USA) can be used to detect PPV-D in 1:100 (*w*/*v*) diluted infected plant tissue crude extracts [37].

Highly sensitive and specific antibodies are critical for the developments of effective serological detection assays [38,39]. In this study, we prepared two super-sensitive and specific MAbs (13H4 and 4A11) using purified PPV virions as the immunogen. Both MAbs can react with PPV in infected apricot tree leaf crude extracts specifically, but not with the extracts infected with additional five plant viruses. The dot-ELISA developed in this work can detect PPV in apricot tree leaf crude extracts diluted up to 1:5120 (*w*/*v*, g/mL). Surprisingly, the detection limit of this dot-ELISA was ~26 times higher than that of RT-PCR. In addition, a highly specific and sensitive CGICS using both prepared MAbs was developed in this study. The developed CGICS can easily monitor PPV in apricot tree leaf tissues but had no cross-reaction with the other five tested virus pathogens including PNRSV, CMV, PVA, PVY, and EAPV, and uninfected plant tissues. The sensitivity analysis indicated that the CGICS can be used to monitor PPV in crude extracts diluted up to 1:6400 (*w*/*v*), equivalent to 9.6 µg of PPV-infected leaf tissues (Figure 5b). Unexpectedly, the detection limit of the developed strip is the same as that of the conventional one-step RT-PCR.

Analyses of 22 field-collected samples further confirmed the usefulness of these two methods and showed that PPV is now widely present in apricot trees in six Chinese cities. Consequently, we recommend the newly developed dot-ELISA and/or CGICS for large-scale onsite PPV detection. Because CPs of PPV Chinese isolates share the high aa sequence identity with that of PPV-D and other PPV strains [6], and PPV in the field-collected apricot leaf samples was accurately detected through RT-PCR using the PPV conserved or PPV-D strain specific primers, we speculate that the developed dot-ELISA and CGICS in this study may be able to detect PPV-D and other strains. We also consider that these two methods can help orchard farmers to eradicate PPV-infected orchards.

In conclusion, we have prepared two super-sensitive and specific MAbs, and used them to develop super-sensitive, reliable, and easy-to-use dot-ELISA and CGICS for PPV detection. We strongly recommend these two methods for PPV field surveys, certifications of PPV-free stone tree materials, and phytosanitary inspections.

## Data Availability

Not applicable.
